# Practical recommendations for fertility preservation in women by the *Ferti*PROTEKT network. Part I: Indications for fertility preservation

**DOI:** 10.1007/s00404-017-4594-3

**Published:** 2017-11-24

**Authors:** A. N. Schüring, T. Fehm, K. Behringer, M. Goeckenjan, P. Wimberger, M. Henes, J. Henes, M. F. Fey, M. von Wolff

**Affiliations:** 1 UKM Kinderwunschzentrum, Department of Gynaecology and Obstetrics, University Hospital of Münster, Albert-Schweitzer Campus 1, D-11, 48149 Münster, Germany; 2 Department of Gynaecology and Obstetrics, University Hospital of Düsseldorf, Düsseldorf, Germany; 3 Department I of Internal Medicine, University Hospital of Cologne, Cologne, Germany; 4 Department of Gynaecology and Obstetrics, TU Dresden, Dresden, Germany; 5 Department of Women’s Health, University of Tübingen, Tübingen, Germany; 6 Centre for Interdisciplinary Clinical Immunology, Rheumatology and Auto-inflammatory Diseases and Department of Internal Medicine II (Oncology, Hematology, Immunology, Rheumatology, Pulmology), University of Tübingen, Tübingen, Germany; 7 Department of Medical Oncology, Inselspital and University of Berne, Berne, Switzerland; 8 Division of Gynaecological Endocrinology and Reproductive Medicine, University Women’s Hospital, Berne, Switzerland

**Keywords:** Fertility preservation, Breast cancer, Hodgkin’s lymphoma, Borderline ovarian tumour, Cervical cancer, Rheumatic diseases

## Abstract

**Purpose:**

Most guidelines about fertility preservation are predominantly focused on scientific evidence, but are less practically orientated. Therefore, practically oriented recommendations are needed to support the clinician in daily practice.

**Methods:**

A selective literature search was performed based on the clinical and scientific experience of the authors, focussing on the most relevant diseases and gynaecological cancers. This article (Part I) provides information on topics that are essential for the fertility preservation indication, such as disease prognosis, disease therapy and its associated risks to fertility, recommending disease-specific fertility preservation measures. Part II specifically focusses on fertility preservation techniques.

**Results:**

In breast cancer patients, fertility preservation such as ovarian tissue and oocyte cryopreservation is especially recommended in low-stage cancer and in women < 35 years of age. In Hodgkin’s lymphoma, the indication is mainly based on the chemotherapy regime as some therapies have very low, others very high gonadotoxicity. In borderline ovarian tumours, preservation of fertility usually is achieved through fertility sparing surgery, ovarian stimulation may also be considered. In cervical cancer, endometrial cancer, rheumatic diseases and other malignancies such as Ewing sarcoma, colorectal carcinoma, non-Hodgkin lymphoma, leukaemia etc., several other factors must be considered to enable an individual, stage-dependent decision.

**Conclusion:**

The decision for or against fertility preservation depends on the prognosis, the risks to fertility and individual factors such as prospective family planning.

## Introduction

**Fig. 1:**
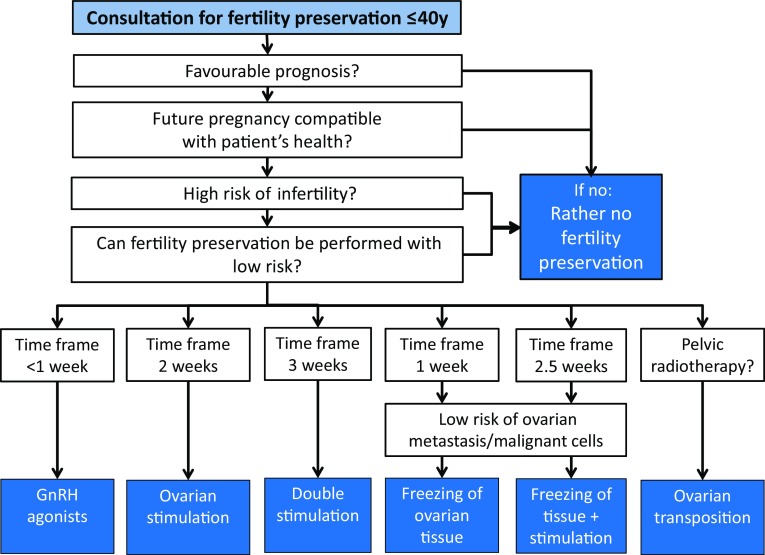
Decision tree for fertility preservation: criteria to decide for or against fertility preservation in women

Fertility preservation techniques have become an established part of oncology, rheumatology and many other areas. Several guidelines and recommendations have been published in Europe [1], the United States [2] and elsewhere. These guidelines mainly focus on scientific evidence, but are less practically orientated. The *Ferti*PROTEKT network, a network and society of physicians and biologists specializing in fertility preservation in Germany, Austria and parts of Switzerland already published practical recommendations [3]. These recommendations have been updated, focusing on indications for fertility preservation, as well as on fertility preservation techniques in women who require therapies which can potentially lead to ovarian failure. As the topic has become too broad for one single paper, we have prepared two articles. This first article (Part I) provides disease-associated information, which is required for recommending fertility preservation procedures; a second article (Part II), also published in this journal, provides information about fertility preservation techniques. We aim to focus on topics that are essential in deciding for or against fertility preservation such as disease prognosis, disease-specific therapy and associated risks to fertility and to recommend disease-specific fertility preservation measures (Fig. 1).

## Breast cancer

### Prognosis

The prognosis of breast cancer is influenced by the tumour stage, intrinsic subtype and genetic classification. Stages II, III and IV display a mortality rate of 20, 44 and 66%, respectively in women with breast cancer < 40 years [4]. Triple negative breast cancer or a luminal B-type tumour are associated with an adverse prognosis [5]. While the overall 10-year survival rate in all women with breast cancer is 86%, the prognosis of younger patients < 35 years is significantly lower, as aggressive subtypes and negative prognostic factors are more frequent in young women [6]. In young women with breast cancer diagnosed at age < 35 years, it has been shown that the survival rate decreases by 5% for each year of lower age at diagnosis [7].

### Risks to fertility

The chemotherapy-related risk of premature ovarian insufficiency (POI) is influenced by age, body mass index, the type and duration of therapy. After six cycles of CMF, the risk of amenorrhoea is 33 and 81% in patients < 40 and ≥ 40 years of age, respectively [8]. Lower rates of persisting amenorrhoea were observed for newer therapy regimes such as AC-, ACT-, FAC- and FACT. In women < 30 years, the risk of amenorrhoea was 10–20%, but 13–68% for women > 30 years [9] (Table 1).

**Table 1:** Risk to fertility by gonadotoxic agents and regimes. Modified from [1, 113, 114]

Risk category	Risk of permanent amenorrhoea	Agent/regime
High risk	80%	HSC-TX with cyclophosphamide/TBI or cyclophosphamide/busulfan
		External beam radiotherapy including the ovaries
		BEACOPP escalated (≥ 30 years)
		6× CMF, CEF, CAF, TAC (≥ 40 years)
		Procarbazine
		Chlorambucil
Intermediate risk	40–60%	BEACOPP escalated (< 30 years)
		6× CMF, CEF, CAF, TAC (30–39 years)
		4× AC (≥ 40 years)
		4× AC or EC → Taxanes
	30%	Monoclonal antibody: bevacizumab
	12–54%	MTX (cumulative risk increased in repeated treatment of autoimmune disorders)
Low risk	< 20%	ABVD (≥ 32 years)
		4–6× CHOP
		CVP
		AML therapy (anthracycline/cytarabine)
		ALL therapy (multi-agent)
		6× CMF, CEF, CAF, TAC (≤ 30 years)
		4× AC (≤ 40 years)
Very low or no risk	–	ABVD (< 32 years)
		Methotrexate
		Fluorouracil
		Vincristine
		Tamoxifen
Unknown risk	–	Monoclonal antibodies: trastuzumab, cetuximab
		Tyrosine kinase inhibitors: erlotinib, imatinib

*HSC*-*TX* hematopoietic stem cell transplantation, *TBI* total body irradiation, *CMF* cyclophosphamide, methotrexate, fluorouracil, *CEF* cyclophosphamide, epirubicin, fluorouracil, *CAF* cyclophosphamide, doxorubicin, fluorouracil, *TAC* docetaxel, doxorubicin, cyclophosphamide, *BEACOPP* doxorubicin, bleomycin, vincristine, etoposide, cyclophosphamide, procarbazine, *AC* doxorubicin, cyclophosphamide, *EC* epirubicin, cyclophosphamide, *MTX* methotrexate, *ABVD* doxorubicin, bleomycin, vinblastine, dacarbazine, *CHOP* cyclophosphamide, doxorubicin, vincristine, prednisone, *CVP* cyclophosphamide, vincristine, prednisone, *AML* acute myeloid leukaemia, *ALL* acute lymphatic leukaemia

While adjuvant endocrine therapies are not gonadotoxic per se, the long duration of therapy poses a significant risk to fertility because of the natural decline in the ovarian reserve during therapy. Therefore, the increased age at the time when endocrine therapy is completed needs to be considered, if fertility preservation measures are discussed [10]. However, endocrine therapy may be interrupted for pregnancy after 2–3 years and can be continued afterwards, if patients are willing to accept a possibly increased risk for recurrence [11].

### Risk of ovarian metastasis

The risk of ovarian metastasis is increased in higher tumour stages with peripheral metastases, in inflammatory and in lobular breast cancer. In tumours with negative lymph nodes, the risk of ovarian metastasis is low. Even in younger patients in higher disease stages and positive lymph nodes, the risk of ovarian metastasis at diagnosis seems to be low, but limited data are available [12–14]. In a study with 2648 young women with breast cancer of whom 2.4% had the diagnosis of ovarian metastasis, the median latency up to diagnosis of ovarian metastasis was 49 months [15]. In a small series using ovarian tissue from 13 women with advanced breast cancer, no metastases were observed after xenotransplantation into SCID mice [16].

The risk for women with BRCA1- and BRCA2-positive breast cancer of developing primary ovarian cancer is 15–65% [17]. In BRCA1- and BRCA2-positive women with prophylactic salpingo-oophorectomy, the ovaries showed occult primary ovarian cancer in 6 and 2%, respectively [18].

### Fertility preservation measures

GnRH agonists have previously been suspected to impair the benefit of chemotherapy in hormone-sensitive breast cancer by causing ovarian suppression. However, as recent studies starting the endocrine ovarian suppression simultaneously with the (neo) adjuvant chemotherapy have questioned such a negative effect [19], this treatment option can be considered even in hormone-sensitive breast cancer.

Ovarian stimulation with oocyte cryopreservation is possible and appears to be safe [20]. If this is discussed with a patient shortly after diagnosis, the time interval before adjuvant chemotherapy is usually sufficient to allow single or double ovarian stimulation [21].

In hormone-sensitive breast cancer, controlled ovarian stimulation should be discussed individually with the patient and the oncologist. If stimulation is performed, estradiol levels should be reduced by aromatase inhibitors (or tamoxifene) and by GnRH agonist triggered ovulation without impairing clinical pregnancy rates and live birth rates in cryopreservation cycles [20, 22–24] and oncological follow-ups [25].

Ovarian tissue cryopreservation appears to be safe, especially in low tumour stages. The stage-dependent risk for ovarian metastases should be discussed with the patient. Ovarian cryopreservation should not be performed in stage IV breast cancer. In patients with BRCA1- and BRCA2-positive breast cancer, ovarian cryopreservation appears to be possible. After completion of family planning, transplants should be removed.

### Summarized recommendations

- Fertility preservation is recommended in women with breast cancer with a good prognosis, with a moderate to high POI risk and/or age > 35 years at the time of expected pregnancy.
- In hormone-insensitive breast cancer, GnRH agonists, ovarian stimulation for oocyte cryopreservation and ovarian tissue cryopreservation can be offered.
- In hormone-sensitive tumours, GnRH agonists and ovarian stimulation for oocyte cryopreservation should be discussed individually.
- If the time interval before chemotherapy is < 2 weeks, e.g., in a neo-adjuvant situation, stimulation is not an option and ovarian tissue cryopreservation should be considered.
- A combination of fertility preserving measures, and freezing the ovarian tissue followed by ovarian stimulation with or without GnRH agonists can be offered.

## Hodgkin’s lymphoma

### Prognosis

Prognosis in Hodgkin’s lymphoma (HL) is age-related with high 15-year survival rates of 94, 91 and 87% in age groups of 18–29, 30–39 and 40–49 years, respectively [26, 27]. Prognosis also depends on disease stage, risk factors and therapeutic response.

### Risks to fertility

Early stages are treated with ABVD chemotherapy, for intermediate stages, the German Hodgkin Study Group recommends 2×BEACOPP escalated plus 2×ABVD and for advanced stages, 4–6×BEACOPP escalated. In other countries, ABVD remains the standard also for advanced HL. BEACOPP escalated is associated with significantly higher gonadotoxicity than ABVD [28] and this effect is age-dependent. After 8×BEACOPP escalated, the rate of amenorrhoea was 51.4% in women aged < 30 years and 95.0% ≥ 30 years [29]. Comparing the gonadotoxic effects of ABVD and BEACOPP escalated, post-treatment serum AMH was 2.2 vs. 0.1 µg/l at age 18–29 years and 0.7 vs. 0.0 µg/l at age 30–45 years [26]. While 90% of patients with early stages of HL who received ABVD ± 2×BEACOPP reported regular menstruation within 1 year of chemotherapy, amenorrhoea persisted in 25% of the 25 years old and in 50% of the 30 years old patients 4 years after 6–8×BEACOPP escalated [26] (Table 1). Radiotherapy in HL exerts an additional negative effect on fertility, when the ovaries are involved [30] (Table 2).

**Table 2:** Radiotoxicity and ovarian insufficiency. Modified from [96, 112]

Ovarian effects of radiotherapy	Ovarian radiotherapy dose (Gy)
No relevant effects	≤0.6
No relevant effects < 40 years	≤1.5
Depletion of follicle pool by 50%	2.0
Risk of ovarian insufficiency 60% (15–40 years)	2.5–5.0
ESD 0 years (at birth)	20.3
ESD 10 years	18.4
ESD 20 years	16.5
ESD 30 years	14.3
ESD 40 years	6.0

The ESD is defined as the radiotherapy dose, which reduces the ovarian follicle pool to less than 1000 follicles in 97.5% of women [112]

*Gy* gray, *ESD* effective sterilizing dose

### Risk of ovarian metastasis

Ovarian metastases were not detected in the four studies, including patients with advanced HL [14, 31, 32]. However, a case report observed HL within ovarian tissue in one patient in stage IIIb with hepatic involvement and pelvic lymphoma [33]. It can be concluded that the overall risk of ovarian metastasis is very low.

### Fertility preservation measures

GnRH agonists and ovarian tissue cryopreservation are options for fertility preservation. Ovarian stimulation and oocyte cryopreservation is feasible, if the time interval before chemotherapy is sufficient to allow for stimulation. HL affecting the mediastinum can occasionally pose a risk for complications during intubation, making laparoscopy for ovarian cryopreservation impossible. Alternatively, if time is sufficient, ovarian stimulation can be offered, as transvaginal oocyte aspiration does not require intubation.

### Summarized recommendations

- Fertility preservation is recommended in women < 40 years with high POI risk (e.g., 6×BEACOPP escalated).
- In women with a low or moderate POI risk (e.g., 2×ABVD or 2×ABVD plus 2×BEACOPP escalated) fertility preservation can be considered.
- GnRH agonists, ovarian stimulation with oocyte cryopreservation and ovarian cryopreservation are adequate options for fertility preservation in HL.
- Laparoscopy for ovarian cryopreservation may not be possible in HL, if a mediastinal tumour impairs intubation.
- A combination of fertility preserving measures, GnRH agonists, and freezing of ovarian tissue followed by ovarian stimulation may be an option, if gonadotoxic risk is high, prognosis is good and time is sufficient.

## Borderline ovarian tumour and epithelial ovarian cancer

### Prognosis

Borderline ovarian tumours (BOT) are mainly diagnosed at an early stage with favorable prognosis, characterized by the absence of invasive peritoneal implants [34]. The 10 year survival rate is 97% for all FIGO stages, for Stage III and IV it is 90% [35].

Epithelial ovarian carcinomas are often detected at advanced stages with a 5-year survival rate of 42%. In FIGO I, the 5-year survival rate is > 90%. The main prognostic factors are post-operative tumour mass and stage of disease, while others are age, constitution, grading, histologic type and guideline adherence during therapy.

### Risks to fertility

Fertility in ovarian tumours is mainly compromised by the surgical procedure, but fertility sparing strategies exist. In epithelial ovarian cancer, chemotherapy can pose additional risks for fertility.

In BOT, guidelines require complete tumour removal, bilateral salpingo-oophorectomy and surgical staging, because the post-operative remaining tumour is the main prognostic factor. Chemotherapy is not indicated in BOT. In early epithelial ovarian carcinoma (FIGO IA, unilateral, G1), surgery involves complete tumour removal, bilateral salpingo-oophorectomy, hysterectomy, omentectomy, peritoneal biopsies and pelvic and para-aortic lymphadenectomy. Chemotherapy is indicated in FIGO > IA, G1.

### Fertility preservation measures

In unilateral BOT, the contralateral adnexa can be preserved. If both ovaries are involved, organ-preserving cystectomy is often feasible [36, 37]. After family planning is complete, surgery should be completed according to guidelines. Fertility preserving surgery in BOT FIGO I is apparently not associated with a strong increase in oncological risk and fertility preservation is possible [38]. In retrospective observational studies, organ-preserving cystectomy resulted in an increased risk of recurrence [39]. In a large study, relapse rate after ovarian preservation was 12.5% in BOT FIGO I and 44% in BOT FIGO III, but only 13% in case of radical surgery [35]. Another study confirmed an increased relapse risk in the remaining ovarian tissue; however, no significant effect on survival rates could be observed, even when a malignant transformation occurred [40].

In early epithelial ovarian cancer (FIGO IA, unilateral, G1), fertility sparing surgery to preserve the uterus and one healthy ovary is also feasible, after adequate staging and informed consent about associated risks. In well-selected patients, fertility preserving surgery is not associated with a change in the recurrence-free interval or survival rate [41–43]. Exact staging, risk assessment and oncological monitoring until birth are required. Completion of surgery is recommended, after family planning is complete.

Ovarian stimulation for oocyte cryopreservation can be considered in BOT, because of the risk of recurrence and especially before salpingo-oophorectomy is performed [38]. Although it is not known if ovarian stimulation increases the risk of BOT relapse, it has been recommended to restrict the number of cycles [44].

In individual cases of early epithelial ovarian cancer (FIGO IA G1/G2) and after informed consent, in vitro fertilization (IVF) may be an option. However, in a meta-analysis of small studies on IVF after fertility sparing surgery in ovarian carcinoma, a potentially negative effect on the oncological outcome was observed [45].

Ovarian tissue cryopreservation is not recommended in BOT and in epithelial ovarian carcinoma, because of the risk of ovarian metastasis. However, experimental methods such as in vitro growth of ovarian tissue (IVG) and xenotransplantation of ovarian tissue into other species to generate follicles may become future strategies in malignant ovarian tumours.

### Summarized recommendations

- In BOT and in early ovarian cancer FIGO IA G1 after complete staging, fertility sparing surgery to allow for pregnancy is feasible, followed by the completion of surgery after delivery.
- In ovarian cancer FIGO IA G2 after complete staging, fertility sparing surgery and achievement of pregnancy can be considered in individual cases, followed by the completion of surgery and chemotherapy.
- Ovarian stimulation with oocyte cryopreservation can be offered to all patients with BOT, when ovarian reserve is compromised by surgery. Ovarian tissue cryopreservation is not recommended in ovarian carcinoma, because of the high risk of relapse, but can be considered in individual cases if bilateral salpingo-oophorectomy is required.

## Cervical cancer

### Prognosis

The prognosis of cervical cancer is stage-dependent, with a high 5-year survival rate of 93% for FIGO I. If lymph nodes are involved, the 5-year survival rate decreases to 20–60%, depending on the localization of metastases. Other important prognostic factors are lymphangiosis, haemangiosis, grading, histology and infiltration of resection margins.

### Risks to fertility

Fertility in treatment of cervical cancer can be compromised by the surgical procedure and by radiochemotherapy. Organ-preserving cone-biopsy or large loop excision of the transformation zone (LLETZ) is performed for carcinoma in situ. While stages FIGO IA1, FIGO IA2 and selected cases of FIGO IB1 < 2 cm can also be treated with fertility sparing procedures, cervical carcinoma FIGO IB1 ≥ 2 cm requires radical hysterectomy with bilateral salpingectomy, which is not compatible with fertility. In cervical adenocarcinoma, bilateral oophorectomy is recommended due to a potential risk of ovarian metastasis; however, the ovaries can possibly be preserved in stage FIGO < IB2.

In the case of combined radiochemotherapy, the detrimental effect on the ovaries is determined by the total and local radiotherapy dose in an age-dependent manner. The use of platinum as a radio-sensitizer in radiochemotherapy causes a potentiation of gonadotoxic effect. A radiotherapy dose of 14.3 Gy will cause complete ovarian insufficiency (sterilization) in 97.5% of women at age 30 [46] (Table 2). Craniolateral transposition of the ovaries should be considered.

Radiotherapy to the uterus in cervical cancer involves a total dose of 40–50 Gy and a boost of 9 Gy as a combination of percutaneous radiotherapy and brachytherapy. A uterine dose of more than 45 Gy is not compatible with future pregnancies [47] (Table 3).

**Table 3:** Clinical effects of radiotherapy to the uterus. Modified from [47]

Radiotherapy during childhood has a more harmful effect on the uterus than during adulthood
Radiotherapy to an adult uterus during total body irradiation (TBI) with 12 Gy is associated with an increased risk of miscarriage, premature birth and low birth weight
After radiotherapy to the uterus with a dose > 25 Gy during childhood, pregnancy is not advisable
After radiotherapy to the uterus with a dose > 45 Gy during adulthood, pregnancy is not advisable

*Gy* gray, *TBI* total body irradiation

### Risk of ovarian metastasis

Ovarian metastases in early stages of cervical carcinoma without risk factors are infrequent, however, in adenocarcinoma in young women, the risk is significantly increased compared to squamous cell carcinoma (8.2 vs. 0.4%, respectively) [48–50].

### Fertility preservation measures

In cervical carcinoma in situ, cone-biopsy or LLETZ do not impair fertility, when the surgical technique aims at preserving cervical function during future pregnancy [51]. In microinvasive carcinoma FIGO IA1 with one risk factor and in FIGO IA2 without risk factors, cone-biopsy is eligible for fertility preservation, if a complete resection of the tumour is achieved (R0). Oncological results are comparable to hysterectomy [52, 53].

In FIGO IA2 plus one risk factor and in FIGO IA1 plus two risk factors, staging which confirms negative lymph node status is required to allow fertility sparing radical trachelectomy including cerclage [54, 55]. Long-term outcome appears comparable to radical hysterectomy [56, 57]. In FIGO IB1 < 2 cm and no risk factors, radical trachelectomy is also possible in selected cases.

For FIGO IB1 ≥ 2 cm, radical hysterectomy with bilateral salpingectomy is indicated and preservation of the uterus is not possible. While preservation of the ovaries is possible in early squamous epithelial carcinoma, in adenocarcinoma, which carries a higher risk for ovarian metastases, this may be individually decided for FIGO < IB2 [58].

When cone-biopsy or trachelectomy are performed for fertility preservation in cervical cancer instead of radical hysterectomy, an increased risk of relapse must be discussed with the patient. In addition, an increased risk of miscarriage and premature birth must be considered [57, 59–62]. Downstaging by neo-adjuvant chemotherapy in advanced cervical cancer for uterus preservation has been described, but is currently considered experimental [63].

Craniolateral ovarian transposition can be performed before radiochemotherapy in selected cases. However, the surgical procedure itself can be associated with a reduction in the ovarian reserve [64].

Currently, it is under discussion whether GnRH agonists reduce the gonadotoxicity of chemotherapy. Ovarian tissue cryopreservation can be discussed, but the risk of ovarian metastases must be considered in adenocarcinoma. If legal in the respective country, surrogacy after oocyte cryopreservation could be an option in cases, where preservation of the uterus is not possible.

### Summarized recommendations

- Fertility sparing surgery such as cone-biopsy or LLETZ is recommended in microinvasive cervical carcinoma FIGO IA1 with one risk factor and in FIGO IA2 without risk factors and if R0 resection is achieved.
- In FIGO IA1 with two risk factors and in FIGO IA2 with one risk factor, fertility preserving surgery in the form of radical trachelectomy according to D’Argent is possible, when staging confirms N0.
- In FIGO IB1 < 2 cm, radical trachelectomy with preservation of the ovaries is possible, the increased oncological risk must be individually assessed.
- In FIGO IB ≥ 2 cm, uterus preservation is not possible.
- In cervical adenocarcinoma FIGO < IB2, the ovaries may be preserved in an individual decision.
- Craniolateral transposition of ovaries is an eligible procedure before radiotherapy.
- If the uterus cannot be preserved, ovarian stimulation and oocyte cryopreservation followed by surrogacy is an option, if legal in the respective country.
- Downstaging cervical cancer by neo-adjuvant chemotherapy to preserve the uterus is controversially discussed.

## Endometrial carcinoma and endometrial hyperplasia

### Prognosis

The prognosis in complex endometrial hyperplasia without and with atypia is excellent after progestin therapy and histological follow-up with hysteroscopy and curettage. In early endometrial carcinoma FIGO IA G1/G2, frequent in younger women < 45 years, standard therapy by hysterectomy with salpingo-oophorectomy is associated with a very good prognosis and a 5-year survival rate of 94%.

### Risks to fertility

Therapy with progestin is not gonadotoxic, but delays realization of pregnancy. In endometrial carcinoma, the standard surgical procedure of hysterectomy with salpingo-oophorectomy is not compatible with fertility, but fertility sparing strategies exist for FIGO IA G1. Radiotherapy is indicated in higher stages of endometrial cancer not eligible for fertility preservation.

### Risk of ovarian metastasis

The risk of ovarian metastasis in early endometrial carcinoma appears to be low [65]. Synchronous ovarian cancer may be present, especially in women with Lynch syndrome [66].

### Fertility preservation measures

In complex endometrial hyperplasia without atypia, cyclic progestin therapy is indicated (e.g., 10–20 mg MPA/day), followed by control hysteroscopy and curettage after 3–6 months before pregnancy is achieved. Alternatively, pregnancy is realized first, followed by progestin therapy.

In complex endometrial hyperplasia with atypia, progestin therapy with 100 mg MPA/day with hysteroscopic and histological follow-up after 3 and 9 months is indicated, followed by the realization of pregnancy.

In well-differentiated type I endometrial carcinoma without infiltration of the myometrium FIGO IA G1, hysteroscopy and curettage for tumour removal is possible, followed by progestin therapy for 6–12 months (e.g., 250 mg MPA/day or a progestin containing intrauterine device). Hysteroscopic follow-up and curettage are required every 3 months. After cessation of progestin, the realization of a pregnancy is possible in a limited time interval. After pregnancy, stage-adjusted completion of surgery by hysterectomy and salpingo-oophorectomy is indicated, because of a high recurrence risk [67–70].

The preservation of the uterus and adnexa in endometrial carcinoma is associated with an increased oncological risk, because of inadequate staging. A risk of ovarian metastasis or synchronous primary ovarian carcinoma must be considered, probably being < 1% in early endometrial carcinoma in young women [71, 72]. However, the preservation of ovaries in low-grade endometrial carcinoma does not appear to impair recurrence-free intervals [73, 74].

IVF has been proposed to reduce the time to pregnancy before a completion of surgery [75, 76]. Hormonal stimulation for oocyte cryopreservation appears possible, if stimulated estradiol levels are reduced with an aromatase-inhibitor or an anti-oestrogen and ovulation is induced by a GnRH agonist. Ovarian tissue cryopreservation appears possible in early endometrial carcinoma, because of a low risk of metastases.

### Summarized recommendations

- In endometrial hyperplasia without atypia, cyclic progestin therapy is indicated (e.g., 10–20 mg MPA/day), with follow-up hysteroscopy and curettage after 3–6 months before a pregnancy is achieved.
- In endometrial hyperplasia with atypia, MPA 100 mg/day or a progestin-releasing IUD is indicated, with follow-up hysteroscopy and curettage after 3 and after 9 months, before pregnancy can be achieved.
- In individual cases of progesterone receptor-positive endometrial carcinoma FIGO IA G1, tumour removal by hysteroscopy and curettage is possible for fertility preservation, followed by progestin therapy with 250 mg MPA for 6–12 months with follow-up in three-monthly intervals. The realization of a pregnancy is then possible within a limited time frame. After pregnancy or in case of relapse, completion of surgery is indicated.
- Fertility preservation is not possible in progesterone receptor-negative endometrial carcinoma FIGO IA G1, or in tumours with higher stages or higher grading. In these cases, hysterectomy with bilateral salpingo-oophorectomy is indicated.

## Rheumatic and autoimmune disorders

### Prognosis

Rheumatic and autoimmune disorders cannot be cured; however, life expectancy can be significantly improved when treated adequately.

### Risks to fertility

Ovarian reserve is impaired by cyclophosphamide (CYC), which is used for immune suppression over a limited time interval in highly active disease stages (orally or as an intravenous pulse therapy). While a single treatment cycle appears to have limited gonadotoxicity, repeated cycles are needed to control the disease and may be necessary again throughout the patient’s life, increasing the cumulative CYC-dose. CYC significantly increases the risk of premature ovarian insufficiency (POI) in patients with autoimmune diseases, ranging from 12 to 54% in the literature, depending on age and cumulative CYC-dose [77, 78]. In addition, ovarian reserve can be reduced by the autoimmune disease per se [79–81]. Consultation about fertility preservation in autoimmune diseases, therefore, is recommended before CYC-therapy [82].

### Fertility preservation measures

The following recommendations are mainly based on evidence available for systemic lupus erythematosus (SLE), because limited data exist for other autoimmune diseases.

Female sex steroids are assumed to contribute to the pathogenesis of SLE, which could account for the observed exacerbation of the disease during ovarian stimulation for fertility preservation. In contrast, positive effects on the disorder associated with GnRH agonists suppressing ovarian function have also been observed [83]. In this context, the exacerbation risk of rheumatic disorders during GnRH agonist therapy is low. While their efficacy in fertility preservation for other disorders appears to vary [84], a protective effect of GnRH agonists specifically in autoimmune disorders has been observed [85–88]. A recent consensus recommends GnRH agonists for young women with SLE who are receiving alkylating agents [82].

Ovarian stimulation is associated with a significant exacerbation risk in SLE, which is probably little less present in primary antiphospholipid syndrome (APS) [89]. The risk of venous thromboembolism (VTE) is increased in autoimmune disorders, being the highest in active stages of APS and SLE. When lupus anticoagulant is present, VTE risk is increased sixfold [90]. Data on VTE risk associated with ovarian hormonal stimulation in autoimmune disease is limited. No VTE was observed in a study on 68 stimulated cycles of 19 patients with SLE and APS who received antithrombotic prophylaxis [89]. In active stages of autoimmune diseases, hormonal stimulation for fertility preservation may be considered in individual cases; sufficient VTE-prophylaxis is required [82]. However, especially in connective tissue diseases, hormonal stimulation may not be indicated because of the exacerbation risk.

Cryopreservation of ovarian tissue is an eligible option in autoimmune disorders. A disease-related reduction of the ovarian reserve should be evaluated in advance.

### Summarized recommendations

- Fertility preservation should be recommended in young women with autoimmune disorders, when CYC-therapy is indicated.
- GnRH agonists are an eligible option for fertility preservation in autoimmune diseases.
- Ovarian stimulation and cryopreservation of oocytes can be applied in individual cases, if risk of exacerbation is low and sufficient time is available. Effective thrombosis prophylaxis is required.
- Cryopreservation of ovarian tissue is an option, if ovarian reserve is sufficient.

## Other malignant diseases

### Ewing sarcoma

#### Prognosis

The prognosis in Ewing sarcoma is categorized into risk classes according to the 5-year survival rate (in brackets):

- Standard risk (70–75%): localized tumour, good response to neo-adjuvant chemotherapy.
- High risk (50%): localized tumour, volume > 200 ml, reduced response to neo-adjuvant chemotherapy, pulmonary metastases.
- Very high risk (20–40%): all other.

#### Risks to fertility

Chemotherapy-related premature ovarian insufficiency (POI) in Ewing sarcoma occurs in ≥ 50% of patients. A significantly higher risk of infertility is expected in case of pelvic radiotherapy or haematopoietic stem cell transplantation [91].

#### Risk of ovarian metastasis

Ovarian metastases were observed in single cases [14, 50, 92–94], but were not confirmed in all the studies [95].

#### Fertility preservation measures

GnRH agonists and, if the time interval before oncological therapy is sufficient, ovarian stimulation with oocyte cryopreservation can be considered. Ovarian tissue cryopreservation is possible. A risk of ovarian metastasis must be discussed. Ovarian transposition is possible, if pelvic radiotherapy is performed [96].

### Osteosarcoma

#### Prognosis

The prognosis of osteosarcoma depends on tumour stage with a 5-year survival rate of 20–80%.

#### Risks to fertility

The risk of premature ovarian insufficiency (POI) was reported to affect 6 of 90 women, after chemotherapy (6.6%) [97].

#### Risk of ovarian metastasis

Ovarian metastases are possible, but were not detected in small studies [14, 92].

#### Fertility preservation measures

Comparable to Ewing sarcoma, GnRH agonists and, if the time interval before oncological therapy is sufficient, ovarian stimulation with oocyte cryopreservation can be considered. Ovarian tissue cryopreservation is possible, if the risk of ovarian metastasis is discussed. Ovarian transposition is possible, if pelvic radiotherapy is performed [96].

### Colorectal carcinoma

#### Prognosis

The 5-year survival rate in tumour stages pT1–3 after R0-resection in colorectal carcinoma is 90, 80, 60% and in rectal carcinoma 90, 70, 40%, respectively.

#### Risks to fertility

The chemotherapy-induced risk of premature ovarian insufficiency (POI) is low to moderate, but high if radiochemotherapy is performed [98–100]. A radiotherapy dose of 45–50 Gy causes POI in > 90% of patients with rectal carcinoma.

#### Risk of ovarian metastasis

The risk of ovarian metastasis seems to be low in tumour stages pT1–3 [14, 50].

#### Fertility preservation measures

If chemotherapy is indicated, GnRH agonists, ovarian stimulation for cryopreservation of oocytes, and ovarian tissue cryopreservation are possible options for fertility preservation. Ovarian transposition should be considered, if radiotherapy is performed. Gestational surrogacy after cryopreservation of oocytes or ovarian tissue may be an option, if legal in the respective country.

### Non-Hodgkin lymphoma (NHL)

#### Prognosis

The prognosis in NHL varies greatly and depends on the respective entity of the heterogeneous disease.

#### Risks to fertility

The risk for the ovarian reserve in NHL is determined by chemotherapy and radiotherapy. It can be high with a POI rate of 40–60% in women of age > 35 years for CHOP and VA-CHOP-B [101, 102] (Table 1).

#### Risk of ovarian metastasis

The risk of ovarian metastasis is high in high-grade NHL and Burkitt’s lymphoma [103]. Other types of NHL exhibit a lower, but still clinically relevant risk [14, 32].

#### Fertility preservation measures

GnRH agonists are an option for fertility preservation. Ovarian transposition can be considered, if pelvic radiotherapy is performed. Ovarian stimulation for cryopreservation of oocytes, and ovarian tissue cryopreservation are not recommended, because of the risk of ovarian metastasis.

### Acute lymphoblastic leukaemia (ALL)

#### Prognosis

The 5-year survival rate in children is 80–90%, but much lower in adults with a range of 20–45% depending on the age group [104, 105].

#### Risks to fertility

A moderate to high POI risk is conveyed by chemotherapy in ALL; the risk is very high in autologous or allogenic stem cell transplantation (Table 1).

#### Risk of ovarian metastasis

ALL conveys a high risk for ovarian involvement with leukaemic cells [50, 92, 106].

#### Fertility preservation measures

GnRH agonists are an option for fertility preservation. GnRH agonist-induced amenorrhoea prevents menstrual bleeding during oncological therapy. Ovarian stimulation with cryopreservation of oocytes is often not possible due to the limited time available. Ovarian cryopreservation is experimental because of the high risk of ovarian malignant cells. In vitro growth (IVG) or xenotransplantation may become future strategies.

### Acute myeloid leukaemia (AML)

#### Prognosis

The 5-year overall survival rate is 24–80% in adults depending on the risk group, and 60% in children < 15 years [104].

#### Risks to fertility

The risk of POI conveyed by chemotherapy is moderate to high; in autologous or allogenic stem cell transplantation, it is very high [107] (Table 1).

#### Risk of ovarian metastasis

The risk of malignant cells in the ovaries is high [50, 92].

#### Fertility preservation measures

Comparable to ALL, GnRH agonists are an option for fertility preservation in AML and GnRH agonist-induced amenorrhoea prevents menstrual bleeding during oncological therapy. Ovarian stimulation with cryopreservation of oocytes is often not possible due to the limited time available. Ovarian tissue cryopreservation is experimental because of the high risk of ovarian malignant cells. In vitro growth (IVG) or xenotransplantation may become future strategies.

## Effects of radiotherapy

### Gonadal function

The effects of radiotherapy on the ovaries are difficult to estimate because of individual variation. Table 2 gives an overview of some effects and the respective radiotherapy dose.

### Uterine function

After total body irradiation (TBI) with a median of 10 Gy, a birth weight of < 2500 g was found in approximately 30% of children, compared to 10% of controls without radiotherapy [108]. After direct pelvic radiotherapy, only scarce data are available [109–111]. In a systematic analysis, it was observed that radiotherapy during childhood appears to have stronger detrimental effects on the uterus than in adulthood. Radiotherapy to the uterus in TBI of 12 Gy was associated with a higher risk of miscarriage, premature birth and low birth weight. After a radiotherapy dose of > 25 Gy during childhood, a pregnancy is not advisable; in adulthood, the respective upper limit is 45 Gy [47]. Table 3 shows the effects of different radiotherapy doses on the uterus.
